# The Simian Immunodeficiency Virus Targets Central Cell Cycle Functions through Transcriptional Repression In vivo

**DOI:** 10.1371/journal.pone.0025684

**Published:** 2011-10-17

**Authors:** Carl-Magnus Hogerkorp, Yoshiaki Nishimura, Kaimei Song, Malcolm A. Martin, Mario Roederer

**Affiliations:** 1 ImmunoTechnology Section, Vaccine Research Center, National Institute of Allergy and Infectious Diseases, National Institutes of Health, Bethesda, Maryland, United States of America; 2 Laboratory of Molecular Microbiology, National Institute of Allergy and Infectious Diseases, National Institutes of Health, Bethesda, Maryland, United States of America; Karolinska Institutet, Sweden

## Abstract

A massive and selective loss of CD4+ memory T cells occurs during the acute phase of immunodeficiency virus infections. The mechanism of this depletion is poorly understood but constitutes a key event with implications for progression. We assessed gene expression of purified T cells in Rhesus Macaques during acute SIVmac239 infection in order to define mechanisms of pathogenesis. We observe a general transcriptional program of over 1,600 interferon-stimulated genes induced in all T cells by the infection. Furthermore, we identify 113 transcriptional changes that are specific to virally infected cells. A striking downregulation of several key cell cycle regulator genes was observed and shared promotor-region E2F binding sites in downregulated genes suggested a targeted transcriptional control of an E2F regulated cell cycle program. In addition, the upregulation of the gene for the fundamental regulator of RNA polymerase II, TAF7, demonstrates that viral interference with the cell cycle and transcriptional regulation programs may be critical components during the establishment of a pathogenic infection in vivo.

## Introduction

The CD4+ memory T cell compartment is the primary target of HIV in humans and of SIV in nonhuman primates (NHP); the destruction of this critical subset sets the stage for the pathogenic events leading to AIDS [1]. The targeted loss of CD4+ memory cells occurs in multiple tissues during the acute phase of infection [2], [3], and this loss, especially affecting cells in gut associated mucosal tissue, strongly impacts the long-term outcome of viral infection. Indeed, for NHP, the extent of memory cell destruction during the acute phase predicts long-term survival. This assault on the adaptive immune system not only affects the antigen specific immune responses but also the mucosa associated immune function, i.e., the control of mucosal barrier integrity and the subsequent immune activation by microbial byproducts [4], [5], [6]. Thus, the protection of the immediate CD4+ memory T cell functions is one of the key elements of HIV vaccines [7], [8].

As have been demonstrated by several studies [2], [3], [4], [6] a selective and massive loss of infected CD4+ memory T cells occurs during the acute phase of infection. The mechanism of this depletion is poorly understood: given the extent of the destruction, even resting memory T cells that are not postulated to support productive viral replication are depleted. To explore the range of virus-induced cellular changes that might explain cell death even in the absence of T cell activation-induced viral replication, we assessed the transcriptional events that occur in vivo just prior to and during the peak of viremia after infection with SIV. Using the pathogenic strain SIVmac239, we were able to define transcriptional events in memory cells at a time when a significant fraction of those cells harbor virus.

Typically, determining the effect of HIV on cellular transcription programs in vivo is made difficult by the paucity of infected cells – during chronic infection, typically only one percent or less of CD4 T cells are infected [9], [10], [11], and many of these may have been infected with defective viruses unable to generate spreading infections. However, recent studies of acute SIV infection [2] show that as many as half of all CD4+ T cells can be infected at the peak of the acute infection. Cells isolated at this time point provide a unique window to evaluate the effect of SIV infection on cellular gene activity.

In this study, we used the SIVmac239 infection model, which allows us to assess the virus induced transcriptional changes in the target cells at the peak of acute infection. We used microarray analysis to assess these changes in flow cytometry sorted subsets of peripheral blood CD4+ T cells from infected rhesus macaques in contrast to their paired pre-infection status. We identify both systemic transcriptional changes (i.e., also occurring in uninfected cells), as well as changes selectively occurring in infected cells. Among the latter include factors central to cell cycle transition, demonstrating that viral interference with the cell cycle is a critical component during the establishment of a pathogenic infection in vivo.

## Results

### SIV mac239 specifically targets CD4+ central memory cells

**Figure 1:**
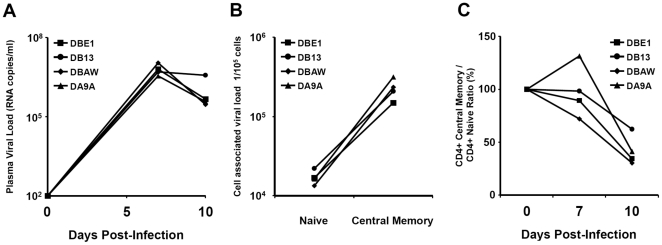
The peak of infection occurs at day 7 post infection. The plasma viral load assessed by a quantitative PCR measurement of viral RNA copies in sampled peripheral blood (A). The major viral production is attributed to CD4+ central memory cells, which constitutes the main target for the CCR5 tropic SIV mac239. At day 10 post infection the cell associated viral load is 13 fold higher in the central memory cells compared to the naive CD4+ cells, as assessed by quantitative PCR for integrated gag DNA (B). A preferential selective loss of central memory cells occurs over the course of the infection. Over half of the central memory cells are lost at day 10 post infection, illustrated here as the percent change in the ratio of central memory cells over naive CD4+ T cells; assessed by flowcytometry (C).

SIVmac239 is a highly pathogenic virus that selectively targets CD4+ central memory cells through obligate use of the CCR5 co-receptor. Four Rhesus Macaques were infected with SIVmac239. The peak of infection occurred at day 7 post infection as suggested by the plasma viral load (Figure 1a). At this time point, we confirmed the selective targeting of memory T cells by quantifying the cell-associated viral load in different subsets (Figure 1b). By day 10 post infection, memory cells showed a 13-fold higher level of gag DNA than naïve CD4 cells, and averaged 2.3 DNA copies per cell. It was previously estimated that at the peak of infection around 30–60 percent of the memory cells are infected and that 80% of these infected cells are eliminated during the next few days [2]. Indeed, in agreement with published data, the relative number of CD4+ central memory cells (as a portion of naïve CD4+ cells) at day 10 post infection dropped 60% (Figure 1c). This suggests that the targeted loss of this subset could be ascribed largely to direct viral infection. Importantly, phenotypic analysis shows there is no (up to day 10) or minor increase (days 14 and later) in the activation state (expression of HLA-DR or CD38) or proliferation state (expression of Ki-67) of the CD4 T cell compartment [12], [13]. At these time points, a majority of infected cells are still in a classical “resting” state.

**Figure 2:**
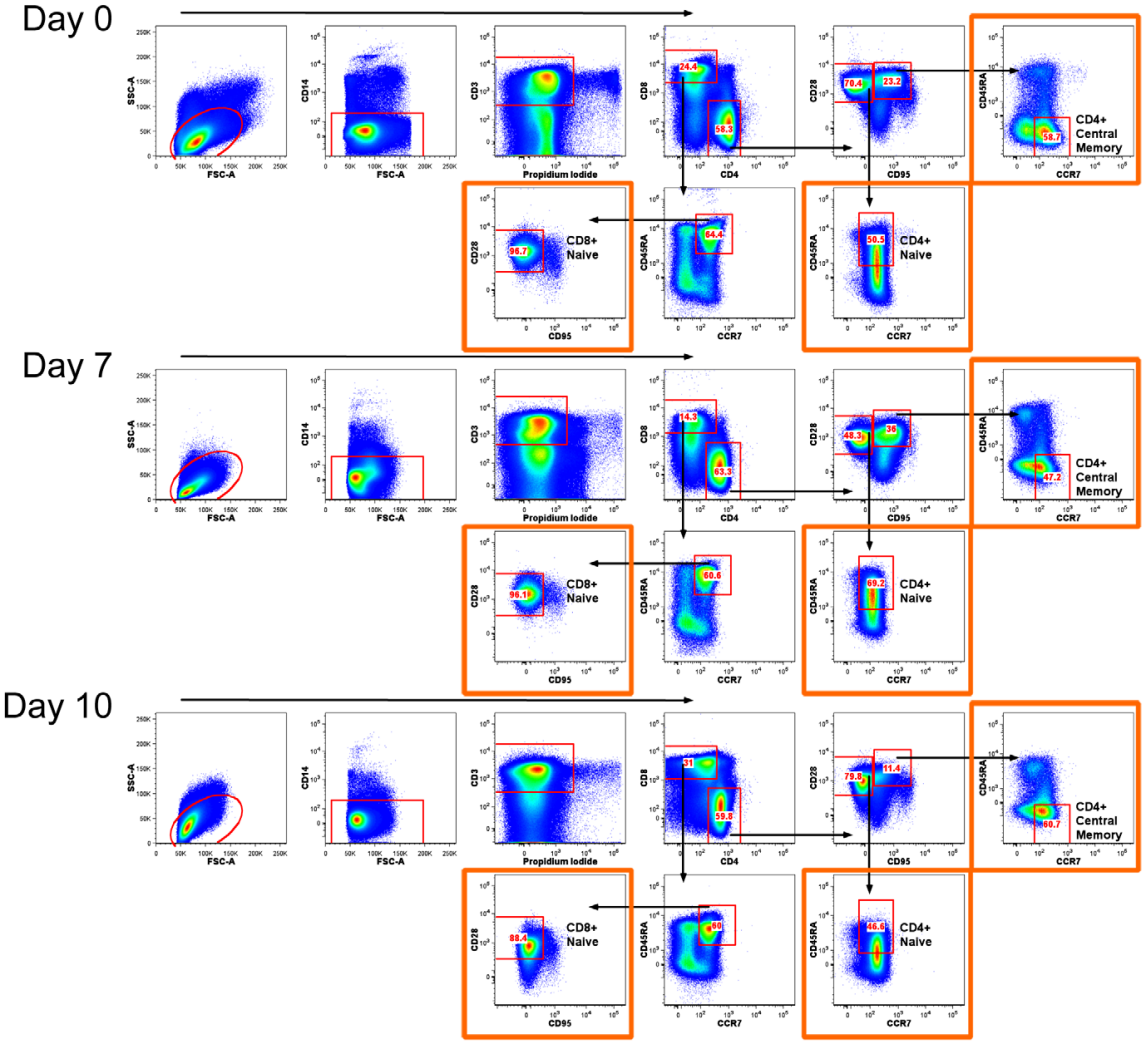
Cell sorting scheme. Flowcytometric cell sorting was used to isolate T cell subsets from each animal at day 0, and at day 7 and day 10 post infection. Subsets of naive and central memory CD4+ T cells and naive CD8+ T cells were sorted using markers to selectively isolate viable CD14− CD3+ cells subsetted as CD4+ CD28+ CD95− CD45RA+ CCR7+ naive CD4+ T cells and CD4+ CD28+ CD95+ CD45RA− CCR7+ central memory CD4+ T cells and further CD8+ CD45RA+ CCR7+ CD28+ CD95− naive CD8+ T cells (see orange highlighted box). The general phenotypic features defined by these markers were maintained over the course of the infection (day 0 – day 10). Arrows indicates the gating strategy for selecting each subset to be sorted. This is a representative figure for one of the four animals (DA9A). See Figure S2 for the profiles of all 4 animals.

**Figure 3:**
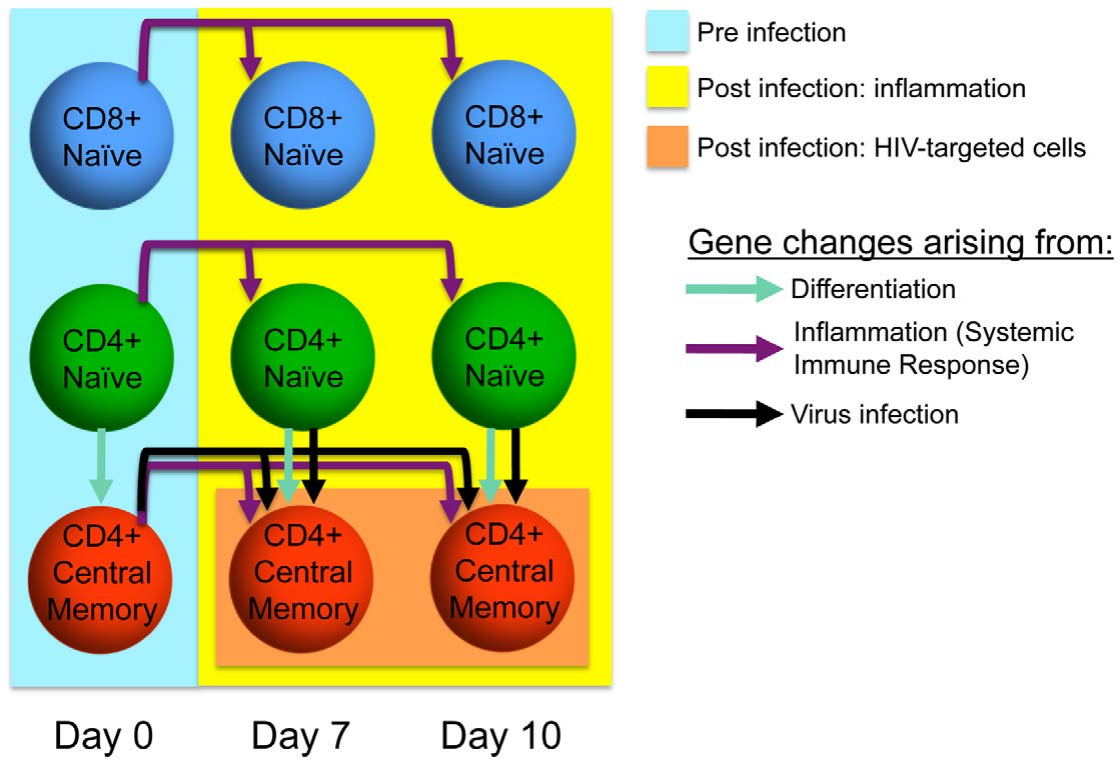
Schematic figure illustrating factors affecting the transcriptional changes in the three different sorted T cell subsets over the course of the infection.

Transcriptional analysis was performed on flow cytometry sorted subsets of naïve and central memory CD4+ T cells along with naïve CD8+ T cells on day 7 and 10 post infection, and compared with paired samples for the same subsets isolated at day 0, i.e., pre-infection (Figure 2). Using this approach we could determine the transcriptional changes that are associated with the virus mediated transcriptional control in the targeted CD4+ memory T cell subset in contrast to those that arise a consequence of a general inflammation (Figure 3). It is important to distinguish the large variety of changes that arise due to a general inflammation (i.e., in trans) from those that are mediated in infected cells in cis. In our model, changes in naïve CD4 and CD8 T cells are mediated in trans; we hypothesize that the remaining changes found in memory CD4 T cells are mediated in cis by viral infection.

### SIV infection induces systemic “Non-Targeted” transcriptional changes

The observation that resting CD4+ cells from viremic HIV+ individuals have a unique gene expression profile in comparison to the same cells from either HIV+ aviremic or HIV^−^ healthy controls suggests that elevated viral replication is leading to altered transcriptional profiles [14]. Indeed, plasma viral levels have been shown to correlate well with immune activation in the host [15] and are likely to affect the transcriptional expression levels seen in CD4+ cells in HIV+ viremic individuals. Although microbial byproducts may be the cause for immune activation in the chronic setting of HIV [5], [16] the contribution of the general inflammation to the pathogenesis of HIV in acute phase has lately been acknowledged [17], [18] and it has been suggested that a pro-inflammatory response may in fact facilitate the infectious process [1], [18], [19], which is an interesting paradox.

In order to assess the scope of such systemic changes we analyzed the gene expression changes that occurs post infection in both the targeted (i.e. CD4+ central memory) and non-targeted (Naïve CD4+ and CD8+) subsets and compared to the pre-infection status. Note that by combining these subsets in this pre- vs. post-infection analysis, transcriptional differences between subsets are largely ignored. Clearly a profound change in the expressional program, comprising thousands of genes, occurs at day 7; declining somewhat towards day 10. An F-test with at a false discovery rate of 0.02 and blocking for any bias from individual differences associated to animal, population and day produced a list of 2,914 genes (1602 up- and 1312 down-regulated) associated to non-targeted changes (Figure S1 and Table S1). Gene ontology and network analysis demonstrated that key features of the list of upregulated genes include interferon responses, antigen presentation and regulation of cell death (data not shown). In the list of down-regulated genes gene ontology analysis indicates that the most significantly changed molecular functions associated to this list are cell death/apoptosis (data not shown).

The suggested inflammation-mediated upregulated gene expression constituted in large what are recognized as interferon-stimulated genes (ISG) [20]. A number of prototypic ISGs were upregulated by the SIV infection (Table 1) and demonstrated a clear response to inflammation [21], [22], [23]. Several of the ISGs are involved in antiviral responses (see Tables 1 and S1), including MX1, MX2, ISG15 (G1P2), ISG20, IFIT1, IFIT3, IFI27, IFI44, ADAR, PML, TRIM22 [24] as well as non-TLR innate immunity regulators such as DDX58, IFIH1 and LGP2 which responds to viral replication [25]. Many of the genes observed in the non-targeted changes have been reported in the NCBI “HIV-1, Human Protein Interaction Database”, and among these are several that are induced by HIV-1 Tat alone [26] (see Table S1). A number of factors in this list are clearly antiviral, but several could also be defined as facilitators of infection, such as AGFG1, AP2B1, CUL5, HIVEP1, IPO7, KPNA1, KPNA5, KPNA6, KPNB1, PML, SMAD3, SMAD4, SP1, SUPT4H1, TAF8, TOP1, XRCC6.

**Table 1:** Prototypic ISGs upregulated in the non-targeted response.

	Number of probes in list	Tat induced (Izmailova et al., 2003)	Antiviral	HIV-1, Human Protein Interaction Database
*CIC*	1			
*EIF2AK2*	3	x	x	x
*EPSTI1*	3			
*GBP1*	7		x	
*GBP2*	3		x	
*ICAM1*	2			x
*IFI16*	1	x		x
*IFI27*	1	x	x	x
*IFI35*	1	x		x
*IFI44*	2	x	x	x
*IFI6* (*G1P3*)	1		x	
*IFIH1*	1		x	
*IFIT1*	1		x	
*IFIT1L*	1			
*IFIT3*	3	x	x	x
*IFITM1*	2		x	x
*IFITM3*	4			
*IRF2*	1			x
*IRF7*	1	x	x	x
*IRF9*	1		x	
*ISG15* (*G1P2*)	1	x	x	x
*ISG20*	1	x	x	x
*LGALS3BP*	1			
*LY6E*	1			
*MX1*	1	x	x	x
*MX2*	2		x	
*OAS1*	3		x	x
*OAS2*	3		x	x
*OAS3*	3		x	x
*OASL*	1		x	
*RSAD2*	2		x	
*SAMD9L* (*C7orf6*)	2			
*SERPING1*	1			
*SP110*	2	x		x
*STAT1*	4	x		x
*STAT2*	2			
*TAP1*	1	x		x
*TNFSF10*	2	x	x	x
*UBE2L6*	1			

### SIVmac239 induces transcription of host genes in the CD4+ central memory target population

The loss of memory cells seen during acute SIV infection [2] significantly exceeds the fraction of activated T-cells normally thought to support productive virus. The depletion of otherwise resting cells thus evokes questions as to the mechanism for this specific depletion. We analyzed the gene expression changes that occur as a result of the infection in the target subset, excluding those changes that were already accounted for above (in the non-targeted cells) and in comparison to the paired pre-infection subsets. These remaining changes include nonspecific (e.g., inflammatory) but selective to CD4 memory cells as well as those changes effected by the virus itself. As noted below, some of these changes are unlikely to be subset-specific inflammatory responses but rather represent plausible virus-mediated changes in transcriptional profiles.

**Figure 4:**
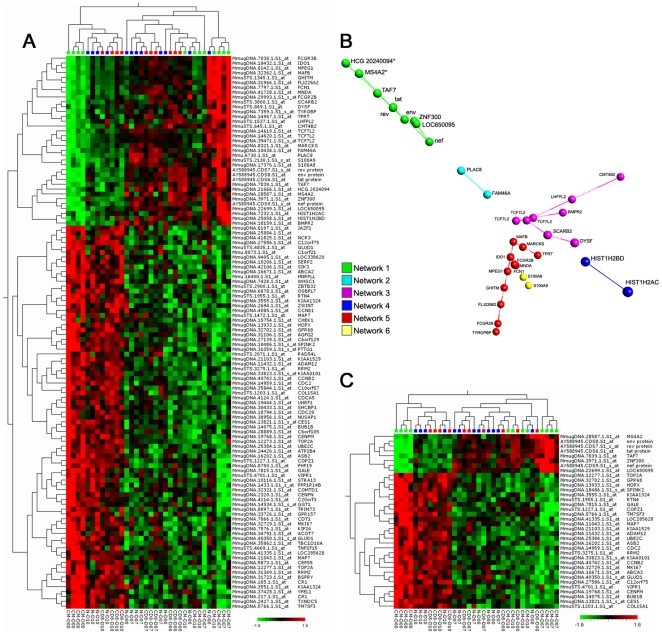
Targeted changes affecting the CD4+ central memory cells. A. Heat map representation of a total set of 113 up and down regulated changes returned by the robust F-test (FDR<0.05). 35 genes are upregulated and 78 genes are downregulated. B. Isomap representation of PCA 1, 2 and 3 of upregulated targeted changes returned by the F-test (FDR<0.05) illustrating how sets of genes forms distinct biologic networks. C. Representation of the more stringent F-test (FDR<0.01) showing a set of 36 up and down regulated genes (8 and 28 respectively). The heat map representations display data normalized to a mean of 0. Hierarchical clustering was performed on the smallest maximum pair wise distance (maximum linkage).

A robust F-test (FDR<0.05 controlling for bias from animal, population and day) of the transcriptional changes occurring in the CD4+ central memory target subset and not in the non-targeted subsets, generated a list of genes of which 35 were up-regulated and 78 were down-regulated (Figure 4a and Table S2). The target transcriptional changes were validated by the expression of SIV genes (env, nef, rev and tat) demonstrating that the observed changes are attributed to the infection of the target subset. In addition to the SIV genes a number of endogenous host genes were part of the list (Figure 4a) and to further understand the potential biologic associations between these genes we applied an Isomap distribution to this gene list; analyzed by principal component analysis [27]. The Isomap analysis is assumed to capture biologic similarities and was used to get a better understanding of the relationships between the genes (Figure 4b). This analysis highlighted several genes that where closely associated to the SIV genes in the isomap 3-dimensional distribution. When increasing the stringency of the F-test to FDR<0.01 (controlling for bias from animal, population and day) the analysis showed a much tighter association of these genes to an infection-mediated transcriptional regulation (Figure 4c). Among the more closely associated endogenous genes where *TAF7* and *ZNF300* along with a number of poorly annotated and described genes. On the other hand both *TAF7* and *ZNF300* are more well-defined genes. The finding of TAF7 is interesting in respect to its documented functional association to Tat [28] and the ZNF300 is likely a transcriptional repressor as it belongs to the KRAB domain containing C2H2 family of zinc binding proteins, which are archetypical repressors [29].

In the list of genes that are down-regulated in the targeted response are, of note, a large number of cell cycle associated genes. 26 out of the 78 down regulated genes are associated with cell cycle functions and several of them are critical for the mitotic program and different levels of checkpoint control functions (Figure 4a and Table S2). These include the two components of the Maturation Promoting Factor (MPF), Cyclin B2 (*CCNB2*) and CDK1/cdc2 (*CDC2*), which is the master regulator of the mitotic program (illustrated in Castedo *et al.* [30]). In addition are found key molecules of the DNA Structure Checkpoint, Chk1 (*CHEK1*), which regulates the initiation of the MPF [30]. Also found are factors controlling transitional events of mitosis such as Cdc20 (*CDC20*), BubR1 (*BUB1B*) and UBE2C (*UBE2C*) which participates in the mitotic checkpoint, regulating the Anaphase promoting complex (APC) and facilitating the destruction of Cyclin B in the metaphase/anaphase of the cell cycle [31]. Several other molecules participating in mitosis are also present in this list, such as, the centromer protein M (*CENPM*) and N (*CENPN*) [32] along with *CCNE1*, *CDCA5*, *CDT1*, *CEP55*, *MKI67*, *NUSAP1*, *PTTG1*, *RAD54L*, *RRM2*, *STRA13*, *TOP2A*, *UHRF1* and *ZWINT*. The concerted regulation of all these functionally related genes suggests a specific transcriptional repression mechanism aimed at modifying cell cycle control in vivo.

### The virus-induced repression of cell cycle genes suggests a virus targeted E2F

In order to understand potential common regulatory targets among the repressed cell cycle genes we analyzed the 1000 base-pair promoter region for shared regulatory sequences among all 26 down regulated cell cycle genes. By comparing to a background set of 41,906 human promoters and running against known cell cycle associated transcription factor binding sites the analysis suggested a highly significant association of E2F matrices among these genes (Table 2). 8 out of the 10 most significant matrix associations were all E2F binding sites, especially E2F1 (Table 2); which suggests a common regulatory mechanism among these 26 genes. Together, these results indicate that infection by SIV targets E2F in a manner leading to large-scale transcriptional changes in cell cycle programming within infected cells.

**Table 2:** Transcription factor binding site analysis for the 26 down regulated cell cycle genes.

Matrix name	P-value	Recognized factors
V$E2F_02	1.75×10^−7^	E2F-1, E2F-2, E2F-3, E2F-3B, E2F-3a, E2F-4, E2F-5, E2F:DP
V$E2F_Q4_01	1.90×10^−6^	DP-1, E2F, E2F-1, E2F-3, E2F-3B, E2F-3a, E2F-4, E2F:DP, E2F:DP:E4
V$E2F1_Q4_01	3.01×10^−7^	DP-1, E2F, E2F-1, E2F-3, E2F-3B, E2F-3a, E2F-4, E2F:DP, E2F:DP:E4
V$E2F_Q6_01	1.15×10^−7^	DP-1, E2F, E2F-1, E2F-1:DP-1, E2F-3, E2F-3B, E2F-3a, E2F-4, E2F-7, E2F:DP, E2F:DP:E4
V$E2F_Q3_01	7.27×10^−7^	DP-1, E2F, E2F-1, E2F-3, E2F-3B, E2F-3a, E2F-4, E2F:DP, E2F:DP:E4
V$E2F1_Q6_01	3.19×10^−19^	E2F-1
V$E2F_Q2	2.82×10^−51^	DP-1, E2F, E2F-1, E2F-3, E2F-3B, E2F-3a, E2F-4, E2F:DP, E2F:DP:E4
V$E2F1_Q3_01	2.42×10^−21^	E2F-1
V$NFY_Q6_01	5.98×10^−5^	CBF(2), CBF-A, CBF-B, CBF-C, CP1, NF-Y, NF-Y′, NF-YA, NF-YA isoform-1, NF-YA isoform-2, NF-YB, NF-YC, NF-YC-3
V$E2F_01	5.43×10^−4^	E2F-1, E2F:DP

*Note: Analysis of the 1000 base-pair upstream region was performed for the 26 down-regulated cell cycle genes. The top 10 binding site matrices are shown.*

## Discussion

To characterize the effects of SIV infection on the cellular transcription in vivo, we isolated T cell subsets from NHP during the acute phase of the infection. Four animals were infected with the highly pathogenic molecular SIVmac239 and the response to the infection was assessed after 7 and 10 days post infection and compared to measurements made pre-infection. We took advantage of the fact that SIVmac239 is CCR5-tropic and infects primarily memory CD4 T cells. This enabled us to specifically address questions as to the specific effect of the virus on its target cells (i.e., memory CD4 T cells) in contrast to effects mediated through generalized inflammation (as revealed by non-targeted cells such as naïve CD4 or naïve CD8 T cells). From the specified time points, we used flow cytometric sorting to purify these subsets of T cells, followed by gene expression profiling, to identify those genetic programs that are selectively modified in the virally-targeted subsets.

A number of studies have employed genome wide approaches to try to understand the complexity of HIV disease, both in vivo [14], [33], [34], [35], [36], [37], [38], [39], [40], [41] and in vitro [42], [43], [44], [45], [46]. However, as HIV/SIV infection generates such a profound innate immune response, transcriptional changes occurring in response to the viral infection risk being masked by transcriptional programs associated to an innate antiviral response. This is particularly true in the setting of chronic infection, where the fraction of infected cells (<1%) is so low that virus-mediated effects are difficult to measure. Many of these studies therefore fail to resolve any viral induced transcriptional changes in endogenous host genes.

Little is known about viral mediated transcriptional regulation of endogenous genes. The predominant way of viral control of host cell events is suggested through protein-protein interactions from factors like Nef, Vpr and Tat [18] and the concept of a virus-mediated transcriptional regulation of endogenous host genes is largely unexplored. However, a number of mainly in vitro studies have suggested that the virus may indeed affect host endogenous transcription [42], [46], [47], [48], [49], but to what extent this control is a direct or indirect virus-mediated regulation is unknown. Many previous studies addressing questions of viral mediated transcriptional control of host genes have also been limited either by the fact that, firstly, in the in vivo setting only a small number of CD4+ T cells are at a given time infected by the virus [9], secondly, a large part of the transcriptional events occurring in an HIV/SIV setting are a consequence of an innate immune- or a cellular antiviral response. We wished to avoid artifacts introduced by ex vivo culture (or the use of cell lines); thus, we used an in vivo model system in which a large fraction of the target population is infected at the peak of infection. We controlled for non-HIV-induced transcriptional changes by comparing the paired non-infected CD4+ subset as well as a CD8+ subset.

### Transcriptional regulation in uninfected cells

It is suggested by *in vitro* studies that HIV viral RNA triggers a TLR7 dependent IFN-α secretion by pDCs [50], [51] leading to a natural response to the viral infection. This pro-inflammatory response will consequently give rise to profound effects on the immune system, which was readily evident in our analysis. However, many secondary effects attributed to HIV-derived factors such as Nef and Tat [52] may also give rise to a multitude of systemic bystander effects. In our analysis, the aggregated response from these indirect effects of SIV infection resulted in a transcriptional change of 2,914 genes, 1,602 upregulated and 1,312 downregulated. Although a TLR7 dependent induction of a type I IFN response is likely to occur, type I IFNs are suggested not to fully account for all ISG changes in HIV [35]. In fact, both gp120 [53], [54] and Tat [26] have been implicated in the induction of an interferon response. Izmailova et al. [26] reports of Tat mediated expression of ISGs in immature DCs and several of the Tat induced ISGs observed in their study are overlapping with our findings of non-targeted changes. Whether this is a functionality of Tat that have evolved to facilitate the infection can only be speculated, but it has been proposed that the pro-inflammatory response induced by the viral infection may promote the infectious process [1], [18], [19] and that factors participating in this response could facilitate the productive infection of target cells. We observed a number of factors in the innate response that have been shown to play important roles for the viral infection of the target cell, factors that are involved in HIV-1 endocytosis, nuclear import, integration, transcription, nuclear export and inhibition of antiviral factors (e.g. *AGFG1*, *AP2B1*, *CUL5*, *HIVEP1*, *IPO7*, *KPNA1*, *KPNA5*, *KPNA6*, *KPNB1*, *PML*, *SMAD3*, *SMAD4*, *SP1*, *SUPT4H1*, *TAF8*, *TOP1*, *XRCC6*). In this respect it is also interesting to note the induction of *IRF2*, which can be linked to the type I interferon response and is exploited by other viral pathogens. IRF2 has recently been demonstrated as a transactivator in the early activation of HPV-16 [55]. HPV-16, akin to HIV-1, is known to regulate early viral transcription by conserved interferon response elements in the viral promotors, binding both IRF1 and IRF2 [56], [57]. As IRF1 has been demonstrated to be required for NFκB transcriptional activity at the HIV-1 long terminal repeat enhancer via interferon response element binding [58] a functional overlap with IRF2 similar to what is seen in HPV-16 could also be suggested for HIV-1. This functional involvement of IRF2 on HIV-1 transcription would thus represent a clear connection to a type I interferon driven regulation of HIV-1.

### Transcriptional regulation in SIV infected cells

From a conceptual point of view the transcriptional control of endogenous host genes can be mediated by either a direct mechanism (i.e. by a direct transcriptional control of the gene, where Tat would be the most likely factor) or an indirect mechanism (i.e. by secondary transcriptional control, through activation or inhibition of transcription factors). Several studies have addressed the impact of endogenous transcription from Tat [26], [47], [48], [59], [60] and Nef [61], [62], [63] in various in vitro systems. It is difficult to draw any clear conclusions from these studies and to what extent the reported gene changes in these studies are also replicated in the in vivo setting. In fact, there is very little overlap between reported genes in any of these studies and the regulation observed in our system. Furthermore, as Tat appears to have a cell cycle regulated transcriptional activity [64], [65] it has been suggested that transcription of endogenous genes regulated by Tat occurs in the G2 phase of the cell cycle, possibly also requiring Vpr induced cell cycle arrest [64]. One of the endogenous genes induced by the virus in our study was *TAF7*, and as TAF7 is involved in the fundamental regulation of RNA polymerase II mediated transcription this is a notable finding. TAF7 is a TATA-binding protein (TBP) associated factor (TAF), which regulates the acetyltransferase activity of TAF1 (a regulator of the transcription preinitiation complex of the basal transcription factor) [66]. It is suggested that TAF7 functions as a check-point regulator of transcription [67] and it is known that TAF7 interacts with the P-TEFb elongation complex, consisting of Cyclin T1 and CDK9, also regulating elongation [28]. TAF7 function also seem to be associated to the G2/M transition [68]. Analogous to Tat, TAF7 also inhibits MHC class I expression in vivo, and the two factors share several functional properties [28], [66], [67]. Together this demonstrates that TAF7 is a transcriptional checkpoint regulator, controlling transcription during the G2/M transition of the cell cycle and having a role in the regulation of MHC class I expression. TAF7 was recently demonstrated to also regulate the acetyl transferase activity of CIITA [69], which is the key factor in TAF1-independent transcription of MHC class I and II in response to infection. One could therefore speculate whether TAF7 is utilized, by the virus, in the control of MHC antigen presentation during infection; and does this also extend to the control of the cell cycle program? In our study we observe transcriptional repression of key cell cycle genes associated to central regulators of the G2/M transition. Our results further suggest that many of the repressed genes are regulated by the E2F-transcription factor family and especially E2F1. As E2F1 itself is transactivated by the basal transcription factor [70], TAF7 mediated acetyl transferase regulation may thus impact on the transcription of the E2F1 target genes. However, another explanation for the down regulation of the central cell cycle regulators may be cytoplasmic sequestration of E2F1. It has recently been suggested that E2F1 may have a role in HIV-induced neuronal damage where it was found that a predominant cytoplasmic localization of E2F1 may account for this neurotoxicity, simply by not being able to transactivate its target genes [71]. The specific down regulation of several E2F1 target genes in the infected CD4+ central memory cells may suggest a similar mechanism in these cells and could also explain the induction of cytotoxic events in infected cells.

In summary, we identified sets of host genes that exhibit significantly altered expression in vivo, during acute SIV infection of rhesus macaques. By purifying T cell populations that are nearly purely uninfected and those cells that are infected at a high rate, we were able to distinguish regulatory effects arising in trans (from inflammatory processes) from those likely arising in cis (effected by virus in the cell). Among the former are large groups of interferon-responsive genes, as expected. The latter group contains a large number of cell cycle-associated genes. This suggests that a critical component of SIV cytopathicity in vivo is a consequence of altered cell cycle regulation. These regulatory events may also underlie the basic mechanism accounting for the productive infection and elimination of a large number of resting, unactivated T cells in vivo.

## Materials and Methods

### Virus and Animals

The origin and preparation of the tissue culture-derived SIVmac239 stocks from molecular clones have been described previously [72], [73]. Rhesus macaques (Macaca mulatta) were maintained in accordance with the guidelines of the Committee on Care and Use of Laboratory Animals [74] and were housed in a biosafety level 2 facility; biosafety level 3 practices were followed. All studies were approved by NIAID and VRC Institutional Animal Care and Use Committees. Four healthy Rhesus Macaques (Macaca mulatta), DB13, DA9A, DBAW and DBE1 (numbered 1, 2, 3 and 4 respectively) were inoculated intravenously with the molecular clone SIVmac239 at 1000 times the 50% tissue culture infectious dose (1000 TCID50). The animals were sampled for peripheral blood at day 7 and day 10 post infection for clinical blood cell count and plasma viral load as well as for T cell isolation by flow cytometry cell sorting.

### Ethics Statement

This study was carried out in strict accordance with the recommendations in the Guide for the Care and Use of Laboratory Animals of the National Institutes of Health. The protocol (LMM32) was approved by National Institute of Allergy and Infectious Diseases, Division of Intramural Research Animal Use and Care Committee (PHS Assurance #A4149-01). Animals were housed and procedures were performed in accordance with all local, state and Federal laws governing in vivo research, in facilities fully accredited by the Association and Accreditation of Laboratory Animal Care International (AAALAC). All procedures were performed under appropriate anesthesia to alleviate pain and minimize suffering as published by the National Institutes of Health Animal Resource Advisory Committee (ARAC).

### Cell associated viral load and plasma viral load

T-cell-associated viral DNA was measured by a quantitative PCR assay for SIV gag using a Applied Biosystems 7900HT instrument (Applied Biosystems), and using SIV gag primers and probe as described previously [2]. Viral-RNA levels in plasma were determined by real-time reverse transcription-PCR (ABI Prism 7700 sequence detection system; Applied Biosystems) as previously reported [75].

### Flow cytometry analysis

EDTA-treated blood samples were stained for flow cytometric analysis using combinations of the following fluorochrome-conjugated mAbs: CD3-PE (clone SP34-2), CD4-PerCP-Cy5.5 (clone L200) CD8-PerCP (clone SK1), CD28-FITC (clone CD28.2), CD95-APC (clone DX2), obtained from BD Biosciences Pharmingen and analyzed by four-color flow cytometry (FACSCalibur, BD Biosciences Immunocytometry Systems). CD4 percentages were multiplied by lymphocyte counts from standard CBC analyses on the same blood draw to determine absolute CD4 counts.

Fluorescence activated cell sorting (FACS) was used to isolate Naïve CD4+, Central memory CD4+ and Naïve CD8+ T cells. The subsets were sorted on the basis of their expression of CD14, CD3, CD4, CD8, CD28, CD95, CD45RA, CCR7 and the viability label Propidium Iodide (PI). The following in house produced reagents were used: CD4-FITC (clone M-T477) CD14-PE (clone M5E2), CD8-QD705 (clone RPA-T8), CD28-QD605 (clone CD28.2) and CCR7-PB (clone 150503). The following reagents were acquired from Beckman Coulter: CD45RA-PETxR (clone 2H4LDH11LDB9) and CD27-APCA700 (clone 1A4CD27); and from BD Pharmingen: CD3-APCCy7 (clone SP34-2) and CD95-APC (clone DX2).

Cells were sorted in an aerosol contained Biosafety level 3 setting on a special order FACS Aria (BD) with Blue, Red and Violet lasers. At least 2×10^6^ cells were sorted from each subset and the sorted cells were collected in 10% serum at 4°C.

### RNA isolation and microarray sample handling

The total RNA was isolated using the RNAqueous®-4PCR isolation kit (Ambion) following the standard protocol. Briefly, cells collected from the cell sorter were pelleted at 860×g, the sheath buffer was aspirated off and the cells were lysed in the supplied lysing/binding solution and subsequently mixed with 64% ethanol. This mix was left over night at −20°C. The RNA isolation was then carried out as stated in the protocol supplied with the kit. A quality analysis of the isolated total RNA was performed on a Bioanalyzer (Agilent) and the material typically had a 260/280 ratio above 1.7. The Affymetrix One-Cycle cDNA Synthesis Kits (Affymetrix) was used to prepare the material for hybridization to the Rhesus Macaque gene chips according to the provided protocol. Hybridized chips were scanned on an Affymetrix Scanner.

### Data analysis

The raw data was quality assessed by principal component analysis, and two samples with outlying behavior were removed from the analysis. Analyses were performed using the Qlucore Omics Explorer (Qlucore AB, Lund, Sweden). All samples passing the quality assessment were imported into Qlucore Omics Explorer as RMA normalized data.

### Annotations

The Affymetrix Rhesus Macaque gene chip genome definitions were fairly rudimentary annotated. At the time of analysis only 25,115 out of 52,865 probes were fully annotated with an official gene symbol (annotations were from Affymetrix Rhesus Macaque na28.annot.20090430). Based on any available annotation data we managed to infer gene symbols for an additional 18,202 probes; 6,913 probes had no annotation at all at the time of analysis. Within the chip 831 probes for genes other than Rhesus Macaque are present. A number of different pathogens are represented such as several herpes virus and hepatitis virus genes along with 17 SIV and 6 SHIV genes. The SIV genes were used as a positive control for infection in our analysis.

### Non-targeted changes

In order to analyze non-targeted changes 11 samples were included in the pre infection control group and 23 samples were included in the post infection group affected by non-targeted changes. The data was analyzed in the Qlucore Omics Explorer (Qlucore, Lund, Sweden) using an F-test with a false discovery rate at FDR<0.05 and controlled for bias from the animal, population and day parameters, which is achieved by eliminating these factors in the general linear model that the software sets up to test the null hypothesis against the alternative hypothesis, where the alternative hypothesis is modeled by the eliminated factors and the test factor.

### Targeted changes

For the analysis of targeted changes all samples targeted by the virus were assessed by the Qlucore Omics Explorer using an F-test at FDR<0.05 and FDR<0.01 and controlling for any bias from animal, population and day. 8 samples constituted the group of targeted samples, consisting of CD4+ central memory cells at day 7 and 10 post infection. The control samples, not infected by the virus included all other subsets and time points and added up to 26 samples in total.

### Functional analysis

Functional, gene ontology and network analysis was performed in the Ingenuity Pathway Analysis database tool (http://www.ingenuity.com/).

### Promoter analysis

Promoter analysis was performed using the BIOBASE ExPlain gene expression analysis system (www.biobase-international.com), mining the TRANSFAC database for transcription factor binding sites. A 1000 base-pair upstream promoter region was analyzed for known cell cycle associated transcription factor binding sites using a background set of 41,906 human promoters.

## Supporting Information

Figure S1 **Heat map representation showing hierarchical clustered transcriptional changes as a consequence of the non-targeted response based on an F-test at a false discovery rate of 0.02 and blocking for any bias from individual differences associated to animal, population and day.** The analysis generated a list of 2,914 genes (1602 up- and 1312 down-regulated) associated to non-targeted changes.
(PDF)

Figure S2 **Cell sorting scheme for all 4 animals at day 0, and at day 7 and day 10 post infection.**
(PDF)

Table S1 **Non-targeted changes following SIV infection.** An F-test at a false discovery rate of 0.02 and blocking for any bias from individual differences associated to animal, population and day produced a list of 2,914 genes associated with non-targeted changes. Tab 1 shows 1,602 upregulated genes and Tab 2 shows 1312 downregulated genes. Tab 1 columns are “Probeset ID”; “Alternate target description” (identifies HIV gene names); “Gene symbol (inferred)” (see Material and Methods for details); “Gene Symbol” (as per original annotation “na28.annot.20090430”); and four columns identifying published characteristics: “Antiviral” (refers to published antiviral properties); “HIV-1, Human Protein Interaction Database” (refers to the NCBI data base); “Facilitators” (refers to published HIV infection facilitating activities), and “Tat induced” (refers to genes reported to be induced by HIV Tat). Tab 2 columns have the same information for genes that are upgregulated following SIV infection.
(XLS)

Table S2 **Targeted changes following SIV infection.** An F-test at a false discovery rate of 0.05 and blocking for any bias from individual differences associated to animal, population and day produced a list of 113 genes associated to transcriptional changes occurring in the CD4+ central memory target subset and not in the non-targeted subsets. Tab 1 shows 35 upregulated genes and Tab 2 shows 78 downregulated genes. Tab 1 columns are “Probeset ID”; “Figure annotation” (refers to relevant annotation); “Gene symbol (inferred)” (see Material and Methods for details). Tab 2 columns also include “Cell cycle” (refers to role in cell cycle, where “+” indicates role in cell cycle and “?” indicated possible role in cell cycle).
(XLS)
