# Supplementary figures and images for: The Simian Immunodeficiency Virus Targets Central Cell Cycle Functions through Transcriptional Repression In vivo
Source: PLoS One. 2011 Oct 17;6(10):e25684. doi: 10.1371/journal.pone.0025684 (PMC3197176; doi:10.1371/journal.pone.0025684)

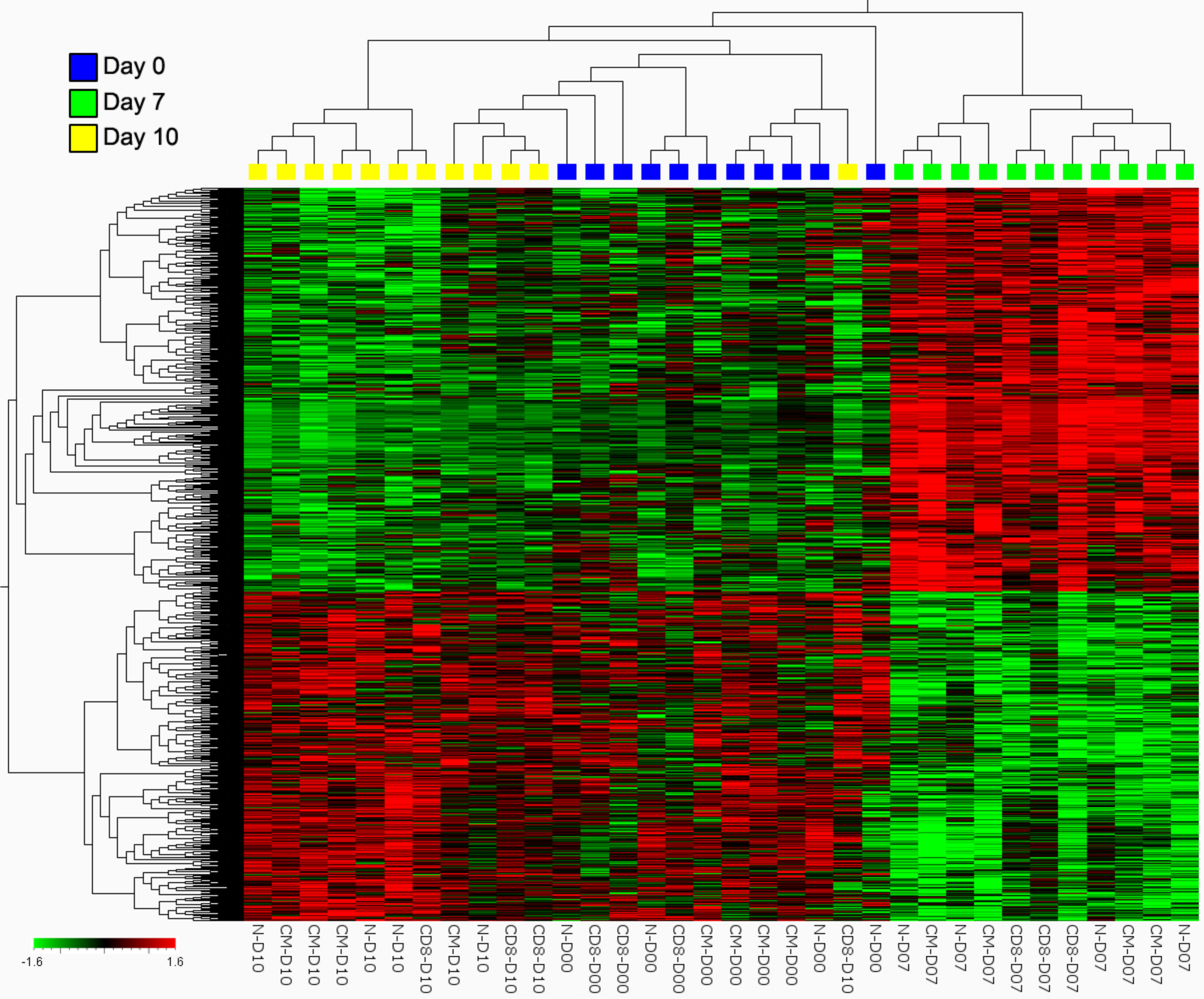

Supplement: Figure S1 — Heat map representation showing hierarchical clustered transcriptional changes as a consequence of the non-targeted response based on an F-test at a false discovery rate of 0.02 and blocking for any bias from individual differences associated to animal, population and day. The analysis generated a list of 2,914 genes (1602 up- and 1312 down-regulated) associated to non-targeted changes. (PDF) [file pone.0025684.s001.pdf]

DA9A

Day 0

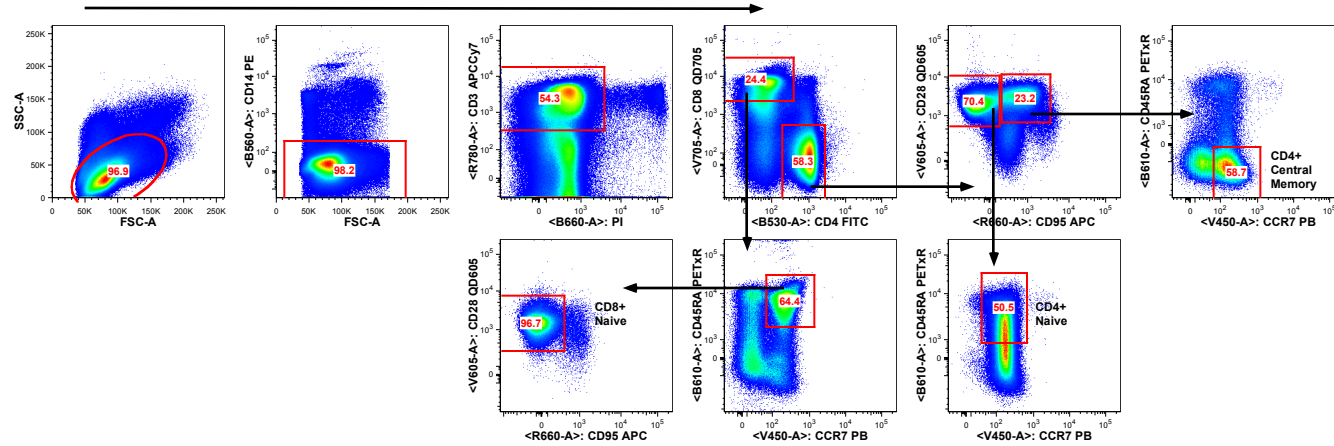

Day 7

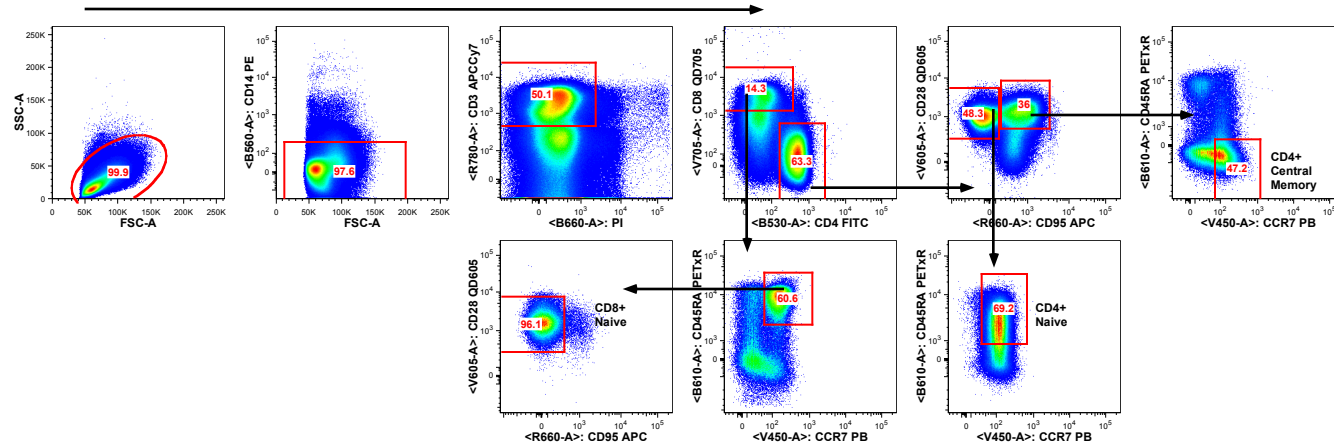

Day 10

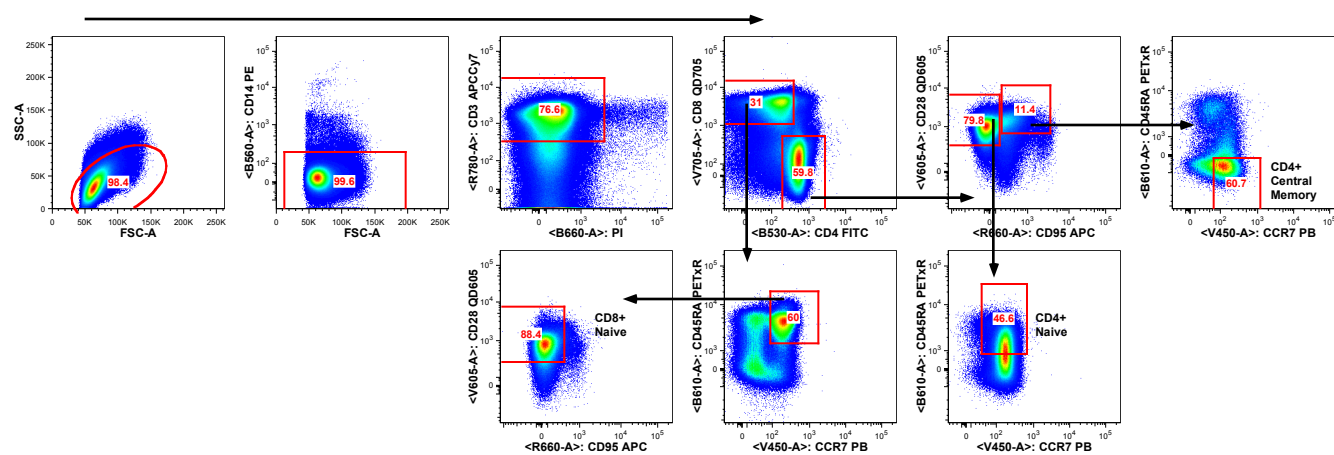

# DBAW Day 0

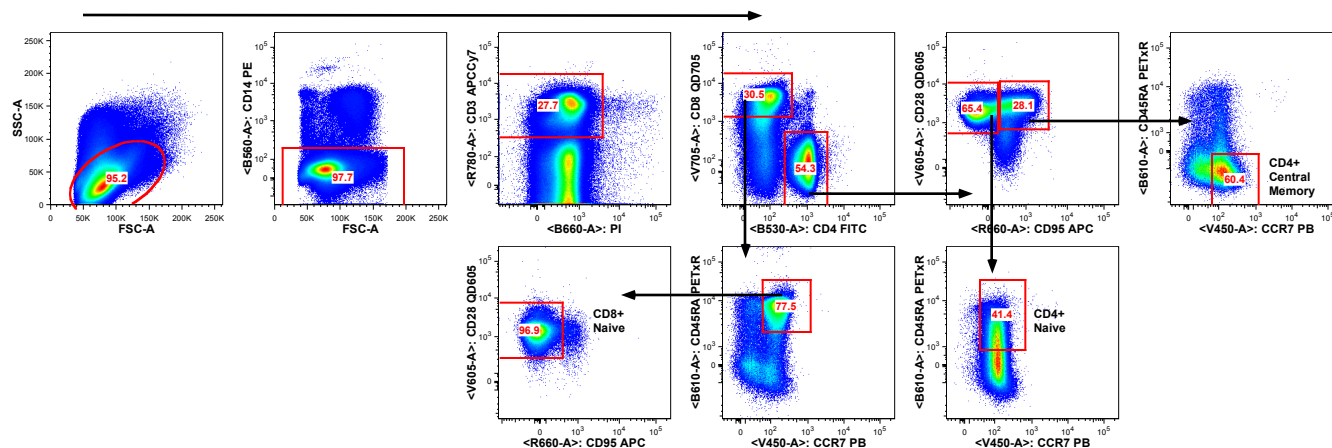

# Day 7

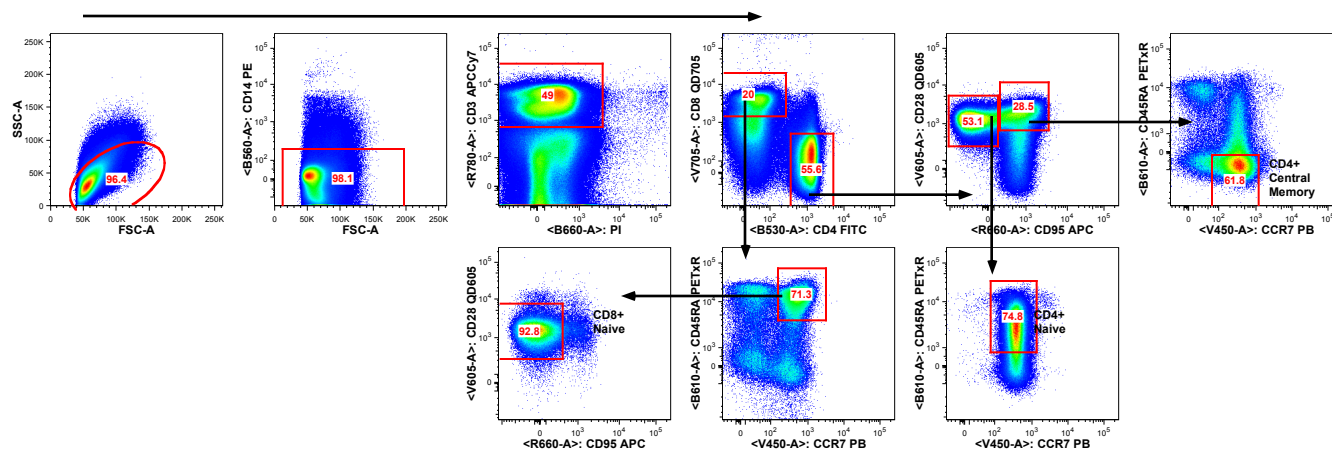

# Day 10

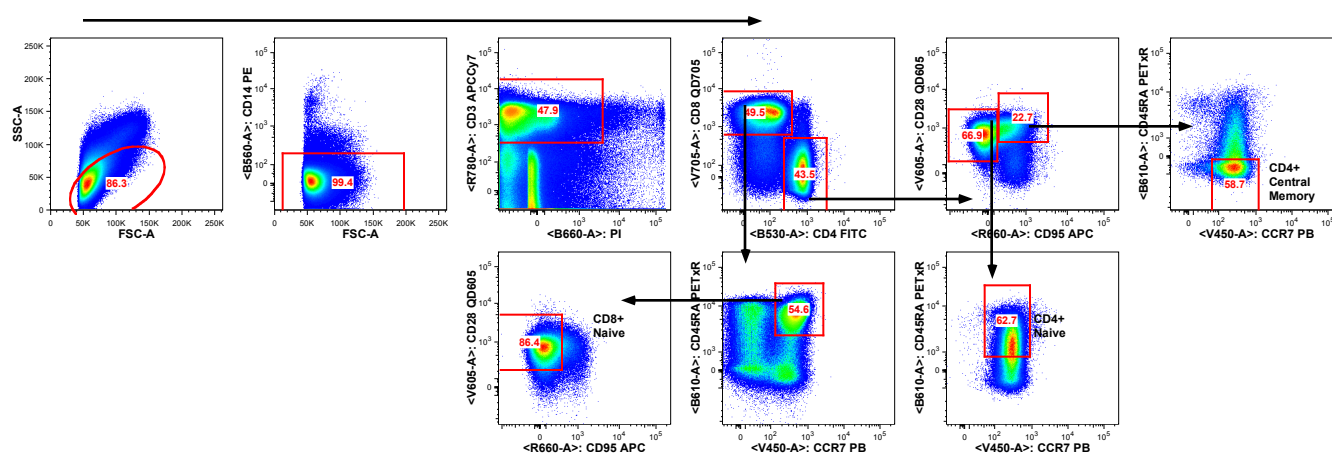

DBE1

Day 0

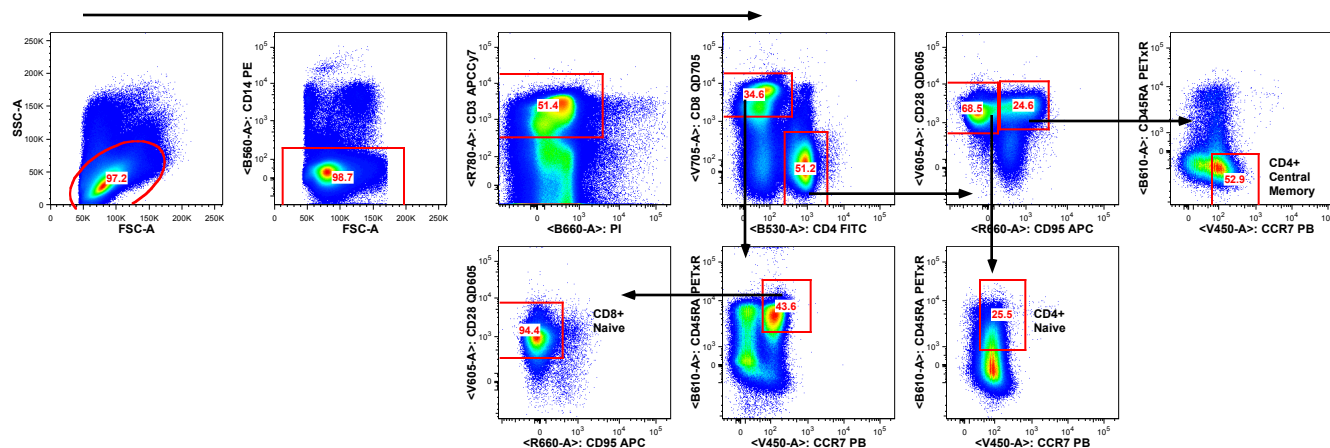

Day 7

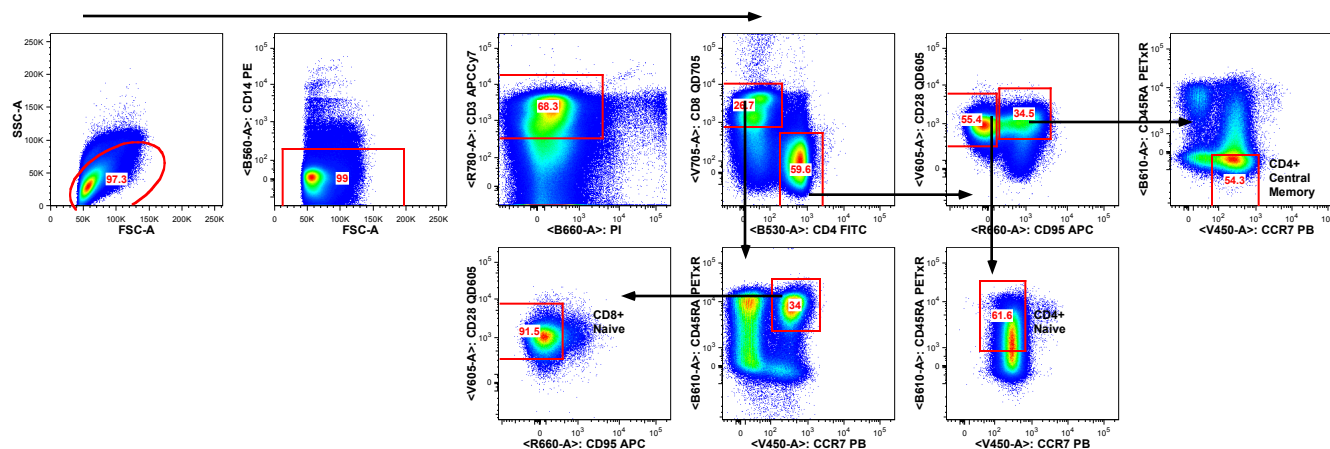

Day 10

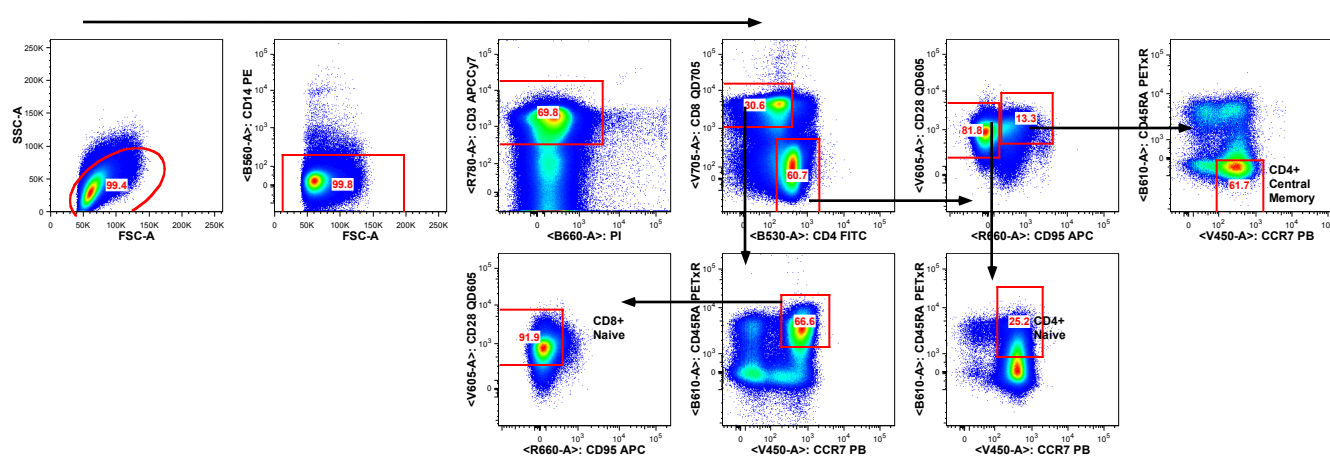

DB13

Day 0

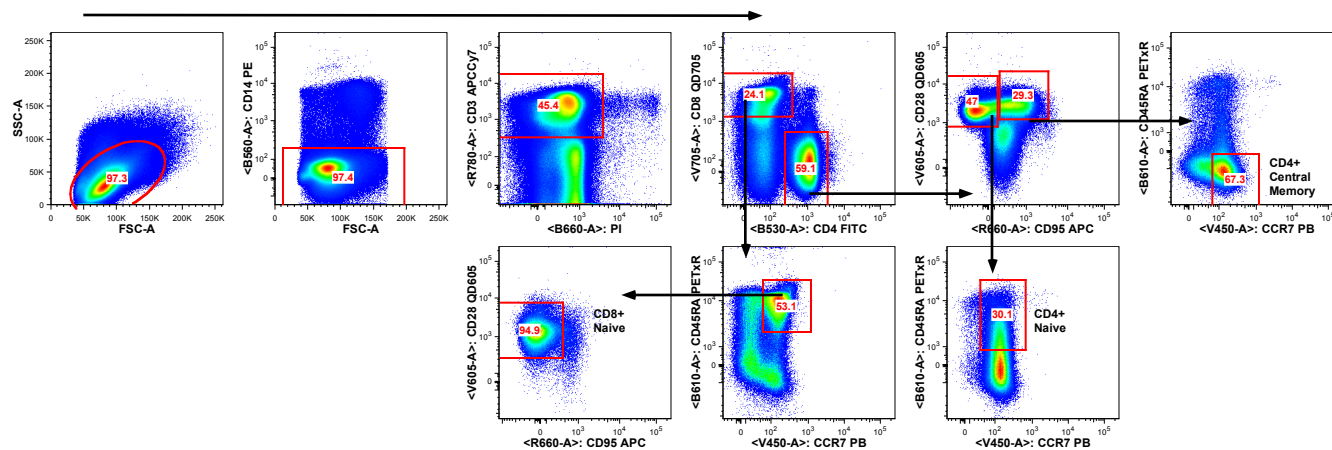

Day 7

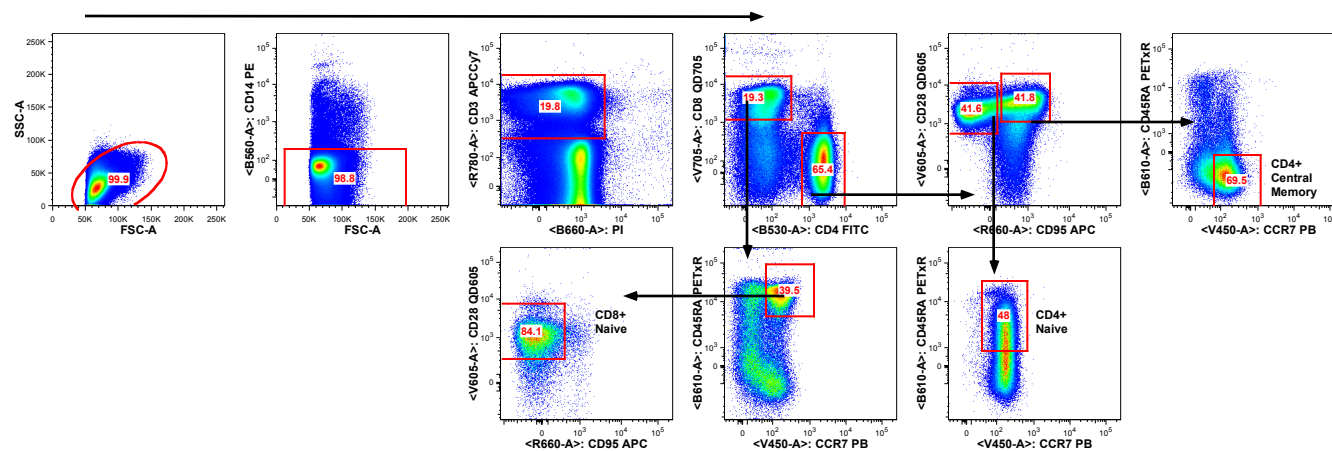

Day 10

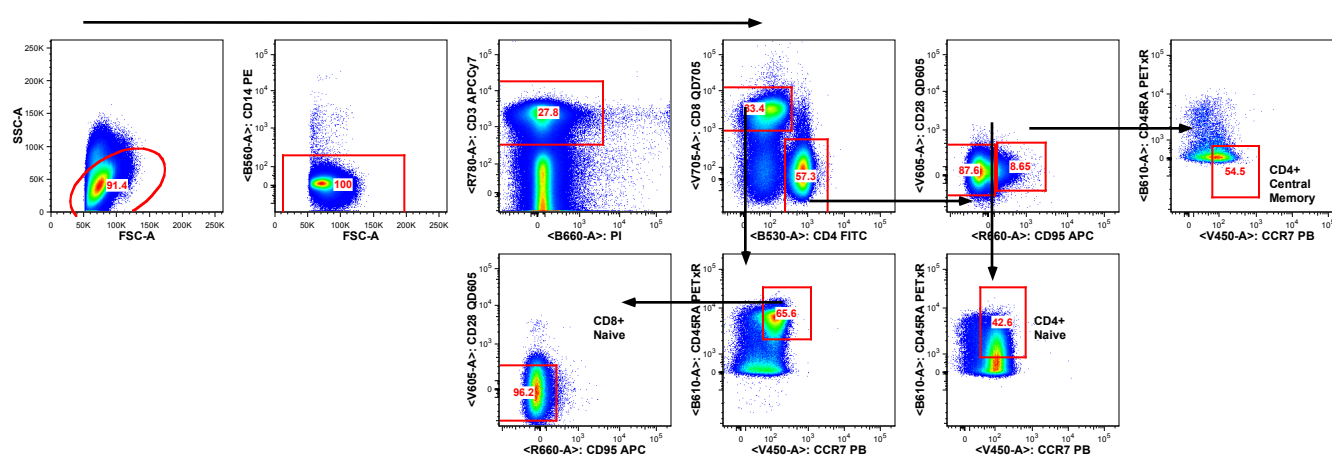

Supplement: Figure S2 — Cell sorting scheme for all 4 animals at day 0, and at day 7 and day 10 post infection. (PDF) [file pone.0025684.s002.pdf]
